# Paleomicrobiology: Revealing Fecal Microbiomes of Ancient Indigenous Cultures

**DOI:** 10.1371/journal.pone.0106833

**Published:** 2014-09-10

**Authors:** Raul J. Cano, Jessica Rivera-Perez, Gary A. Toranzos, Tasha M. Santiago-Rodriguez, Yvonne M. Narganes-Storde, Luis Chanlatte-Baik, Erileen García-Roldán, Lucy Bunkley-Williams, Steven E. Massey

**Affiliations:** 1 Center for Applications in Biotechnology, California Polytechnic State University, San Luis Obispo, California, United States of America; 2 Department of Biology, University of Puerto Rico, San Juan, Puerto Rico; 3 Department of Pathology, University of California San Diego, San Diego, California, United States of America; 4 Center for Archaeological Research, University of Puerto Rico, Rio Piedras Campus, San Juan, Puerto Rico; 5 Department of Biology, University of Puerto Rico, Mayaguez Campus, San Juan, Puerto Rico; University of Illinois, United States of America

## Abstract

Coprolites are fossilized feces that can be used to provide information on the composition of the intestinal microbiota and, as we show, possibly on diet. We analyzed human coprolites from the Huecoid and Saladoid cultures from a settlement on Vieques Island, Puerto Rico. While more is known about the Saladoid culture, it is believed that both societies co-existed on this island approximately from 5 to 1170 AD. By extracting DNA from the coprolites, followed by metagenomic characterization, we show that both cultures can be distinguished from each other on the basis of their bacterial and fungal gut microbiomes. In addition, we show that parasite loads were heavy and also culturally distinct. Huecoid coprolites were characterized by maize and Basidiomycetes sequences, suggesting that these were important components of their diet. Saladoid coprolite samples harbored sequences associated with fish parasites, suggesting that raw fish was a substantial component of their diet. The present study shows that ancient DNA is not entirely degraded in humid, tropical environments, and that dietary and/or host genetic differences in ancient populations may be reflected in the composition of their gut microbiome. This further supports the hypothesis that the two ancient cultures studied were distinct, and that they retained distinct technological/cultural differences during an extended period of close proximity and peaceful co-existence. The two populations seemed to form the later-day Taínos, the Amerindians present at the point of Columbian contact. Importantly, our data suggest that paleomicrobiomics can be a powerful tool to assess cultural differences between ancient populations.

## Introduction

Coprolites are fossilized fecal specimens that give us an opportunity to infer on an extinct organism's diet and intestinal microbiota if the DNA is well preserved. Taphonomic conditions such as a highly biomineralized environment or a rapid decline in the sample's water activity (Aw), induce the fossilization process [1]. It has been widely believed that feces are not well preserved in tropical environments due to the high humid conditions. This may likely be one of the reasons why coprolite studies of indigenous Caribbean and other tropical/subtropical cultures are scarce. However, we obtained human coprolites from Vieques, an island approximately 8 Km off the southeastern coast of Puerto Rico. Located in the Caribbean Sea, 2,020 km from the Equator, the climate in Vieques is generally humid with yearly temperatures ranging from approximately 24 to 28°C.

Puerto Rico is considered an important area for archaeological studies in the Caribbean due to the variety of ancient deposits that have been found on the island. Over 3,000 ancient settlements have been discovered to date, of which, 250 are located in the Island of Vieques and correspond to at least four different ancient cultures that inhabited the island. Among these cultures were the Saladoids and Huecoids, two horticulturalist cultures that coexisted in Vieques (an Island off the coast of Puerto Rico) for over 1,000 years (from 5 AD to 1170 AD) after migrating from South America. Originally from present-day Venezuela, the Saladoids migrated to the island of Vieques by 160 BC and to the main Island of Puerto Rico by 430 BC [2], [3], [4]. While living in this region they maintained their ancestral heritage, as shown by their signature use of white and red painted pottery. However, they also incorporated different traits that they gradually learned/observed from other cultures present on the island. In contrast, little is known about the origins of the Huecoid culture, but they are believed to be originally from the eastern Andes in present-day Bolivia and Peru and are known to have settled in Puerto Rico by at least 5 AD. The Huecoids are characterized by their delicate carvings of semiprecious stones and by their resilience to incorporate material or cultural traits from other cultures [5]. The apparent representation of a pair of Andean condors among their amulets suggests to archaeologists their ancestral residence in the Andes. This is also supported by the possible practice of cranial deformation. Both of these cultures greatly impacted other indigenous cultures present on the island, and are thought to have played a part in the development of the predominant culture colloquially referred to as the “Taínos” [6].

Paleomicrobiological studies have shown marked differences between the microbial communities present in Huecoid and Saladoid coprolites [7]. These studies were performed using Terminal Restriction Fragment analysis (TRFL-P), a technique that, although extremely useful for community profiling, has some intrinsic limitations. For example, its total scope extends to the relatively limited database used for downstream analyses. This intrinsic bias implies that microorganisms not in the database will remain hidden to this type of analysis. Also these analyses are often biased towards the most predominant species, leaving out important information on the rare microbiota present in the sample [8]. In light of these limitations, next-generation sequencing (NGS) represents a more appropriate technique for analyzing these type of samples, mainly due to the high resolving power that characterizes NGS platforms conjointly with bioinformatics tools. Although microbial community profiling when using NGS also has some bias towards the most predominant DNA in the sample, this happens to a lesser extent. Also, NGS is capable of detecting and amplifying previously unidentified/uncultured microorganisms that could be relevant to analysis of the sample's microbial community [9].

The principal aim of this study was to compare these two ancient populations and corroborate whether they present marked differences in their fecal microbiomes due to diet and/or cultural factors. In order to achieve this, we compared the microbiota found in the cores and cortices of coprolites from both cultures using NGS.

## Materials and Methods

### Sample Description

**Figure 1:**
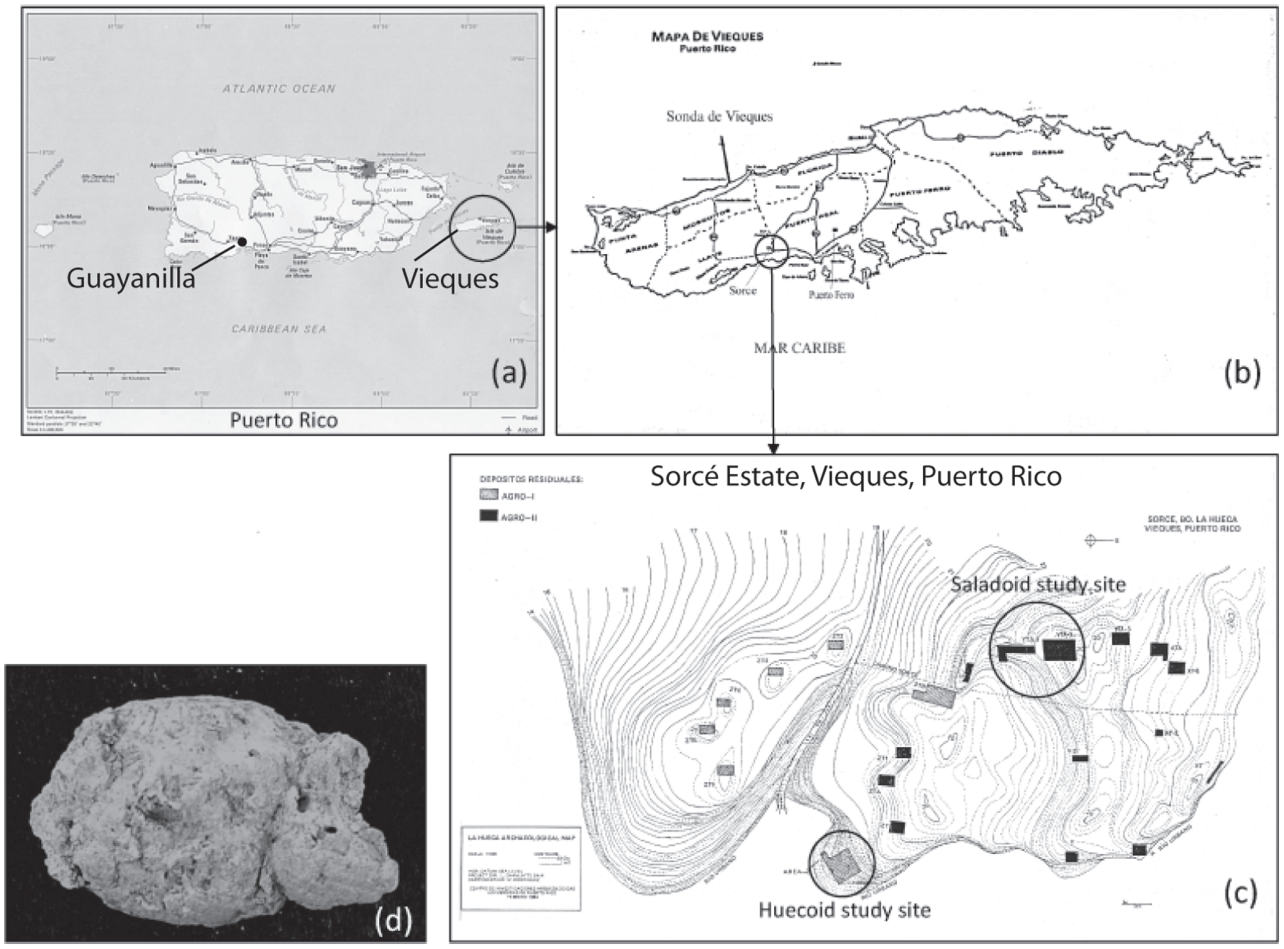
Location and obtainment of coprolites used in this study. Panels (a) and (b) show the sampling sites, located in Sorcé, Vieques, an island off the eastern coast of Puerto Rico. Panel (c) shows the Huecoid and Saladoid archaeological study sites (namely AGRO-I and AGRO-II, respectively). Panel (d) shows a coprolite extracted from these archeological sites.

Coprolites were found in two excavations directed by the archaeologists Dr. Luis A. Chanlatte-Baik and Yvonne M. Narganes-Storde. One excavation site was located at the Sorcé Estate in La Hueca, Vieques, Puerto Rico, the second in Tecla, Guayanilla, PR (**Figure 1**). A total of thirty-four coprolites were used in this study. All thirty-four samples were used for parasitological studies (Huecoid n = 12; Saladoid n = 22). Five of the Saladoid samples used were from Sorce, Vieques and the remaining from Tecla, Guayanilla. In addition, fifteen of the coprolites screened for parasites were also used for microbiome analyses (**Table 1**). Coprolites were provided for this study by Luis Chanlatte-Baik and Yvonne Narganes at the Center for Archaeological Research at the University of Puerto Rico, Rio Piedras Campus. All necessary permits for the collection of samples used in this study were obtained from the Center for Archaeological Research at the University of Puerto Rico, Rio Piedras Campus., complying with all relevant regulations. Repository information, including the nomenclature for precise identification of each specimen containing geographical location, excavation site and archaeological depth for coprolites used in both the DNA and parasitological analyses are described in **Table 1**. Coprolites dated from 180 A.D. to 600 A.D., as indicated by previous ^14^C dating of material obtained from the same or an equivalent archaeological excavation quadrant and depth of each coprolite (e.g. charcoal and mollusk shells) [10] (**Table 1**). ^14^C dating was conducted by Teledyne Isotopes (Westwood, NJ) and BETA Analytic, Inc. (Miami, FL) using standard methods.

**Table 1:** Description of coprolite samples employed in study.

Sample ID	Specimen Number^1^	Culture	Sampling Area	Radiocarbon date
1a	SV_YTA-1_I-5_6– 60 cm	Saladoid	Cortex	335–395 A.D.
1b			Core	
2a	SV_YTA-2_J-22_80 cm	Saladoid	Cortex	270–385 A.D.
2b			Core	
3a	SV_YTA-2_M-25_40 cm	Saladoid	Cortex	230–385 A.D
3b			Core	
4a	SV_Z-W_2.0 m	Huecoid	Cortex	1300–220 A.D.
4b			Core	
5a	SV_YTA-2_I-24_1-1.2 m	Saladoid	Cortex	Circa 385 A.D.
5b			Core	
6a	SV_Z-M_1.2 m	Huecoid	Cortex	Circa 450 A.D.
6b			Core	
7a	SV_YTA-2_I-15_1 m	Saladoid	Cortex	285–375 A.D
7b			Core	
8a	SV_Z-W_1.8 m	Huecoid	Cortex	Circa 245 A.D.
8b			Core	
9a	SV_Z-W_ 1.6 m	Huecoid	Cortex	Circa 385 A.D.
9b			Core	
10a	SV_Z-C_1.8 cm	Huecoid	Cortex	Circa 245 A.D.
10b			Core	
11b	SV_Z-20_2.0 m	Huecoid	Core	Circa 245 A.D.
12b	SV_Z-X_60 cm	Huecoid	Core	470–600 A.D.
13b	SV_Z-L_70 cm	Huecoid	Core	Circa 385 A.D.
14b	SV_ Z-T_S-1_0.9 m	Saladoid	Core	Circa 385 A.D.
15b	SV_YTA-2_H-21_1.2 m	Saladoid	Core	230–385 A.D

1 Prefix SV indicates the sampling site was in the Sorcé Estate (S) the Island of Vieques (V) Puerto Rico. The remaining characters refer to the specific excavation site from which the specimens were obtained (e.g., YTA-1_I-5-6_) and archaeological depth (e.g., 60 cm).

### Sample Handling and Processing

Coprolites selected for DNA analyses were cut in half; one portion was used for DNA extraction and the other for parasitological studies. All sample processing and DNA extractions for the microbiome analyses were performed in an Ancient DNA laboratory where DNA extraction is conducted in class II hoods assigned exclusively for ancient DNA use. Hoods were exposed to UV light for at least 20 minutes before and after every use. Lab coats designated exclusively for ancient DNA use were routinely decontaminated overnight with commercial chlorine (Clorox). Other aseptic measures include the routine decontamination of the working space with chlorine, the use of sterile, baked and autoclaved DNA-free instruments to extract the DNA, as well as gloves. Controls were done *ad-libitum* for the absence of extraneous DNA.

### DNA extraction

To minimize the presence of environmental DNA, approximately 3 mm of the outermost exterior shell of the coprolites was first removed using sterilized brushes as described previously [7]. Approximately 0.25 g of cortex and core samples were separated from each coprolite in the above hoods and DNA was extracted using the PowerSoil DNA Isolation Kit following the manufacturer's instructions (Mo Bio Laboratories, Carlsbad, CA).

### DNA amplification and sequencing

Fifteen coprolites encompassing paired samples of cortex and core (for a total of 30 samples) were sequenced using the Ion Torrent PGM System for sequencing (Life Technologies Corp.) of 16S rRNA gene reads and the Roche 454 FLX Titanium instrument for detection of 18S rRNA genes.

The 16S rRNA gene V4 variable region was amplified using the PCR primers 515f (GTGCCAGCMGCCGCGGTAA)/806r (GGACTACHVGGGTWTCTAAT) [11]. This particular region was selected in order to target both bacteria and archaea present in the samples. PCR amplifications were conducted using a single-step 30 cycle PCR using the HotStarTaq Plus Master Mix Kit (Qiagen, USA) under the following conditions: 94°C for 3 minutes, followed by 28 cycles of 94°C for 30 seconds, 53°C for 40 seconds and 72°C for 1 minute, after which a final elongation step at 72°C for 5 minutes was performed. Sequencing was performed at Molecular Research Laboratory, (www.mrdnalab.com), (Shallowater, TX, USA) on an Ion Torrent PGM following the manufacturer's guidelines. Similarly, the fungal 18S rRNA gene was amplified using SSUfungiF (TGGAGGGCAAGTCTGGTG) / SSUFungiR (TCGGCATAGTTTATGGTTAAG) (Hume et al., 2012). A single-step 30 cycle PCR using HotStarTaq Plus Master Mix Kit (Qiagen, Valencia, CA) was used under the following conditions: 94°C for 3 minutes, followed by 28 cycles of 94°C for 30 seconds; 53°C for 40 seconds and 72°C for 1 minute; after which a final elongation step at 72°C for 5 minutes was performed. All amplicon products from each samples were mixed in equal concentrations and purified using Agencourt AMPure beads (Agencourt Bioscience Corporation, MA, USA). Samples were sequenced utilizing the Roche 454 FLX Titanium instrument and reagents according to manufacturer's guidelines

### Ancient and Extant Sequence analyses

Sequences of extant Amazonian indigenous cultures were obtained from the Short Read Archive (SRA) database (Accession numbers: ERX115092, ERX115316, ERX115218, ERX115130, and ERX115095). The sequences were microbiomes obtained from amplification of the V4 region of the 16S rRNA gene. These sequences were downloaded in FASTA format and used for all comparative studies as described below.

Raw sequence data were prepared for microbiome analysis using QIIME [12]. A total of 3.4 million multiplexed reads from both the 454 and PGM runs were assigned to samples based on their corresponding barcode using *split_libraries.py* using default filtering parameters. 16S rDNA sequences from coprolites were analyzed, individually or merged with modern stool microbiomes. Coprolite 16S rDNAdemultiplexed sequences were sorted based on sample ID using the QIIME script *extract_seqs_by_sample_id.py* and grouped into core and cortex subsets for further analysis. (**Table 2**). *De novo* Operational Bacterial and fungal operational taxonomic units (OTUs) were selected using *pick_de_novo_otus.py* workflow, obtaining a total of n = OTUs. For *18S* data set from QIIME-formatted Silva 111 reference database for Quast et al 2013; (http://www.arb-silva.de/) genetic reference database for eukaryotes for OTU picking and taxonomy assignments (*assign_taxonomy.py*) was used. *16S* and *18S* taxonomy was defined by ≥97% similarity to reference sequences. The phylogenetic composition of the micro-communities present in the samples was characterized using *summarize_taxa_through_plots.py* up to the genus (L7) level.

**Table 2:** Coprolite sequence statistics.

16S SSU						18S Fungi SSU					
Coprolite Core			Coprolite Cortex			Coprolite Core			Coprolite Cortex		
*SampleID*	*Seq Count*	*Mean Length*	*SampleID*	*Seq Count*	*Mean Length*	*SampleID*	*Seq Count*	*Mean Length*	*SampleID*	*Seq Count*	*Mean Length*
1b	112,710	207±87	1a	281,055	210±87	1b.ssu	1,930	457±106	1a.ssu	10,485	463±107
2b	235,943	204±85	2a	112,178	219±83	2b.ssu	3,187	458±106	2a.ssu	3,242	434±101
3b	97,550	227±80	3a	153,768	212±85	3b.ssu	2,295	450±111	3a.ssu	2,021	449±104
4b	302,010	206±86	4a	66,082	218±79	4b.ssu	5,191	462±107	4a.ssu	1,895	410±102
5b	15,788	159±83	5a	311,595	213±86	5b.ssu	5,088	458±106	5a.ssu	5,587	421±98
6b	30,782	227±81	6a	222,080	220±85	6b.ssu	1,510	452±105	6a.ssu	4,761	446±103
7b	226,285	222±82	7a	148,502	206±84	7b.ssu	2,577	469±105	7a.ssu	5,273	445±103
8b	248,994	212±86	8a	254,785	213±84	8b.ssu	8,119	468±109	8a.ssu	9,753	449±112
9b	23,862	207±88	9a	114,920	222±83	9b.ssu	2,886	471±108	9a.ssu	4,429	453±99
10b	18,049	189±96	10a	104,132	219±82	10b.ssu	3,411	460±107	10a.ssu	1,991	440±112
11b	26,308	225±87	11a	ND	ND	MEDIAN	3,037		MEDIAN	4,595	
12b	15,002	213±81	12a	ND	ND						
13b	11,834	221±80	13a	ND	ND						
14b	23,487	208±87	14a	ND	ND						
15b	18,073	201±85	15a	ND	ND						
MEDIAN	26,308		MEDIAN	151,135							

### Alpha and beta diversity

Alpha diversities and rarefaction curves of communities found in coprolite cores and cortices were computed using the *alpha_rarefaction.py* workflow with a custom parameters file that included Shannon statistical analysis. Alpha diversity metrics such as Chao1, which estimates the species richness, Observed_Species, which counts the unique OTUs in a sample and PD_Whole_Tree, which is based on phylogeny, were used for this analysis. Beta diversity distance matrices, UPGMA trees and PCoA plots were computed using *jackknifed_beta_diversity.py*, with default parameters. Distance matrices between sample types were also computed using Primer E v6 software. For comparative purposes, these data were analyzed in parallel with extant fecal microbiomes

### Statistical Analysis

Comparisons between coprolite core and cortex microbiomes were made using Procrustes and Adonis analysis (per mutational multivariate analysis of variance using distance matrices). For Adonis, an unweighted UniFrac distance matrix was used using the QIIME script *compare-categories.py* For Procrustes analysis, the beta diversity of the coprolites' cortices and respective cores were compared using QIIME 1.8. The principal coordinate matrices from unweighted UniFrac PCoA plots from core and cortex samples were transformed using the *transform_coordinate_matrices.py* script and the resulting matrices compared using the script *compare_3d_plots.py.*

Comparison of the microbiomes from coprolites of Saladoid and Huecoid origin were done using the *compare_categories.py* script of QIIME 1.8. For this comparison both the Adonis and Permanova methods were conducted using the unweighted UniFrac distance matrix generated by *beta_diversity_through_plots.py* with 999 permutations.

### Microscopic analysis for Parasite Eggs

A total of 34 coprolites were used to search for parasites in both cultures. One gram of each coprolite was rehydrated in 14 mL of an aqueous solution of trisodium phosphate 0.5% for 72 h [13]. Samples were shaken vigorously, screened through a 1,500 µm mesh separating all macroscopic material from the sample. To the resultant filtrate, 1 ml of 10% acetic acidic-formalin solution per 10 g of filtrate was added (10∶1) to avoid bacterial and fungal growth [14]. The filtered sample was allowed to settle for 72 h after which ten microscope slides were prepared. 50 µL of sediment from each sample was deposited on a slide and mixed with a drop of glycerin. A cover slip was placed on top and the slide was scanned in a serpentine manner covering the whole slide[15], [16]. Each parasite egg and larvae found was photographed and measured at 40× and 60× with a calibrated ocular micrometer. For the lack of a taxonomic key to identify parasite eggs, morphological characters such as projections, shape, and presence of larva inside were used for identification. Other non-parasitic organisms were also photographed. All parasite studies were done at the University of Puerto Rico, Mayagüez, Campus.

## Results

**Figure 2:**
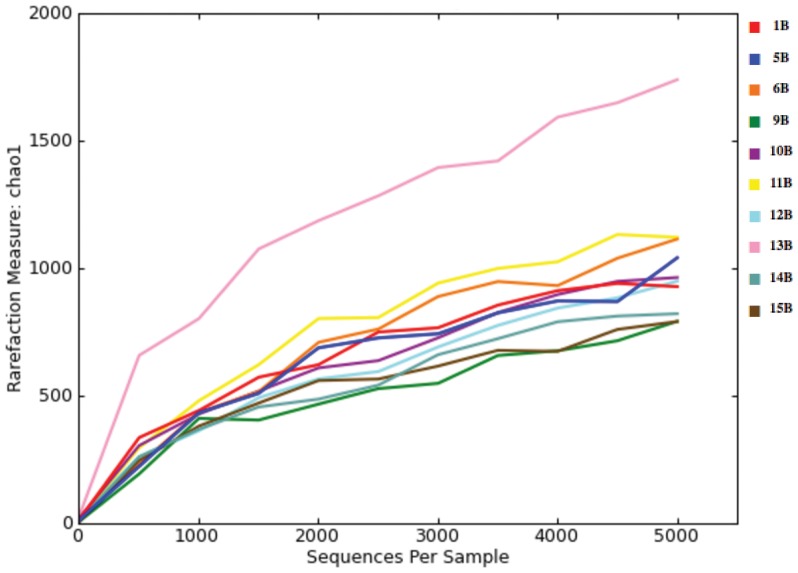
Rarefaction plots the 16S rRNA gene microbiome of the coprolite samples. Rarefaction plots for Huecoid (4B, 6B, 8B, 10B, 11B, 12B, 13B) and Saladoid (1B, 2B, 3B, 5B, 7B, 9B, 14B, 15B) coprolites are shown. Plots were generated using the chao1metic of QIIME 1.8 *alpha_rarefaction.py* with a sampling depth of 5,000. All 15 samples were obtained from the core region (B) of each coprolite.

16S rRNA gene sequences for the coprolites studied ranged from 11,834–281,055 with a median of 26,308 and 151,135 for core and cortex samples respectively (**Table 2**). Fungal 18S rRNA gene sequences ranged from 1,510 to 10,485 with a median of 3,037 for core samples and 4,595 for cortical samples (**Table 2**). Alpha rarefaction plots showed a sampling depth of 5,000, representing approximately 75% of the sample with lowest species count (**Figure 2**). Alpha diversity metrics were consistently higher for *16S* than *18S* in all samples. Diversity indices are depicted in **Table 3**.

**Table 3:** Alpha Diversity Metrics for Coprolite Microbiomes.

Sample ID	Shannon		PD_Whole_Tree		Chao1		Observed_Species	
	16S–V4	Fungi SSU	16S–V4	Fungi SSU	16S–V4	Fungi SSU	16S–V4	Fungi SSU
1a	9.943	4.372	557.945	7.745	14000.189	329.923	10867	234
1b	9.964	3.517	434.365	2.621	11793.849	101.111	8286	56
2a	9.625	2.483	397.655	3.527	11256.698	104.250	7577	68
2b	8.839	2.513	357.141	1.935	9232.645	74.375	6993	53
3a	8.852	2.662	265.672	2.069	6953.633	75.111	5060	54
3b	8.578	3.518	214.855	1.969	5759.892	121.667	4067	70
4a	9.027	ND	224.176	ND	6032.968	ND	4066	ND
4b	9.926	3.189	589.744	2.794	15360.207	128.000	11915	97
5a	10.213	4.623	631.177	6.248	15850.235	243.410	12764	185
5b	8.807	2.360	157.685	1.698	4624.760	91.625	2698	60
6a	9.022	2.436	368.085	2.561	9811.314	87.769	7084	70
6b	6.171	1.995	75.281	0.377	1760.210	21.000	1111	11
7a	9.340	3.525	374.334	5.605	9543.409	167.625	7210	130
7b	8.912	1.046	342.978	1.316	8869.651	66.000	6756	31
8a	10.245	1.951	608.415	3.815	15947.904	248.882	12098	134
8b	9.805	1.033	500.801	3.125	12884.610	180.615	9918	101
9a	8.851	3.358	405.891	4.074	11308.664	252.154	7663	125
9b	8.756	2.257	445.816	1.397	11669.569	72.429	8986	53
10a	8.534	1.701	313.578	1.963	8843.658	105.500	5817	62
10b	8.486	2.394	318.719	2.226	8173.095	80.714	5947	61

### Statistical Analysis

**Figure 3:**
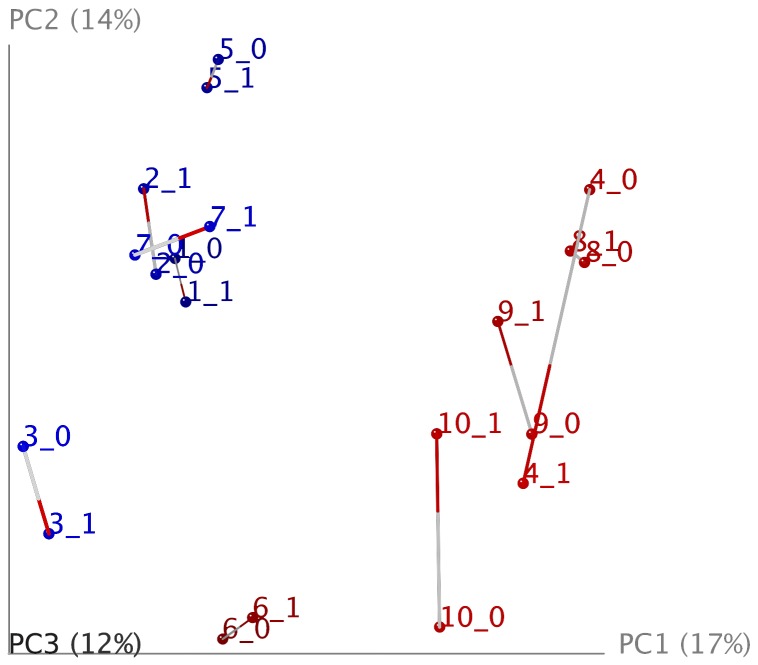
Procrustes analysis compares the 16S microbiome found in the cores and respective cortices of coprolite samples. Samples 6,10, 4, 9 and 8 are of Huecoid origin. Samples 1, 2, 3, 5 ND 7 are of Saladoid origin. Samples identified with number “0” were obtained from coprolite cores and those with the number “1” are from cortical surfaces of the coprolite.

Procrustes analyses of core and cortex samples were conducted as a control study to assess the fecal microbiome in the coprolites and to see any differences from obvious soil contaminants. Procrustes results showed differing beta diversities when comparing the cortices of the samples to their corresponding cores (**Figure 3**). Cortices showed higher proportions of soil-associated microbes (e.g. 65% Actinobacteria and 11% Firmicutes) compared to the coprolite cores (49% Actinobacteria and 6% Firmicutes). Based on these results, all further studies were conducted using core samples exclusively

Similarly, Adonis analysis showed a significant difference between core and cortex microbiomes with an R^2^ of 0.191 (p = 0.001). Adonis and Permanova analyses showed significant differences between the microbiomes of Huecoid and Saladoid coprolites. The Adonis test yielded an R^2^ value of 0.287 and a p value of 0.001. Similarly, the Permanova analysis resulted in a Pseudo-F value of 1.98 (p = 0.001).

### Fecal microbiome in each culture

#### 16S rDNA analysis

**Figure 4:**
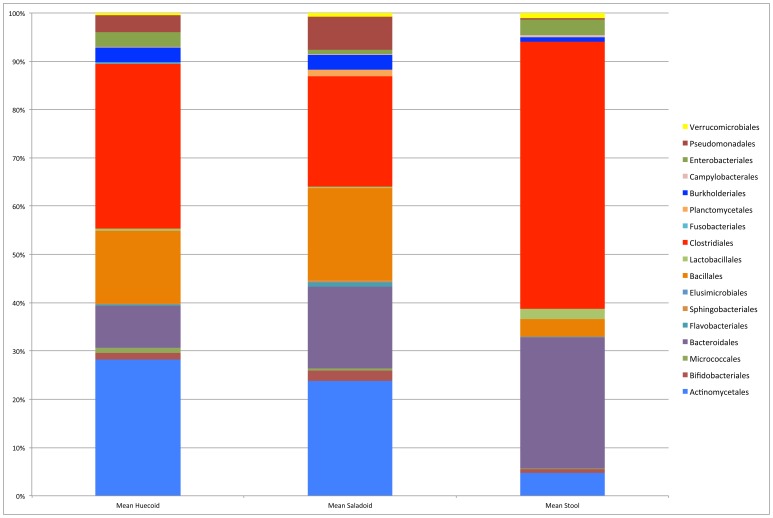
Taxonomic comparison of Huecoid and Saladoid Microbiomes. Figure was generated using summarize_taxa_through_plots.py workflow of QIIME 1.8. Results are illustrated at the Order level. Extant Amazonian stools microbiome was included for comparisons. Mean values for each culture represent taxa obtained from 8 Huecoid coprolite cores, 7 Saladoid samples, and 5 adult, extant Amazonian stools.

Figure 4 illustrates the differences in taxonomic composition of the Huecoid and Saladoid fecal microbiota. Extant Amazonian fecal microbiome was included for comparative purposes. The proportions of key microorganisms showed major variations between cultures, suggesting possible differences in their diets. While the Burkholderiales, Sphingomonadales and Lactobacillales were more represented in both the Huecoid and Saladoid cultures, the Neisseriales and Bacteroidales were more represented in the Amazonian gut.

Table 4 compares the percent similarities (A) and differences (B) in the fecal microbiomes of the Huecoid and Saladoid. For instance, Bacteroidetes were found to be 13% of the Saladoid fecal microbiota, in comparison Bacteroidetes in the Huecoid comprised approximately only 3% of the microbiota. As there are limitations in targeted *16S* and *18S* sequencing and the limited data available in gene databases, some of the microbiota could only be confidently identified at a phylum or class level, while others were identified at the Order and Family level.

**Table 4:** Similarities (A) and differences (B) found in the microbial fecal communities of the Huecoid and Saladoid cultures.

Taxonomy			Huecoid (%)	Saladoid (%)
*Phylum*	*Class*	*Order*		
**(A) % Taxa Similarities**				
Actinobacteria	Nitriliruptoria	-	0.1	0.1
Actinobacteria	Thermoleophilia	-	48.6	53
Bacteroidetes	Flavobacteriia	-	0.1	0.4
Proteobacteria	Gammaproteobacteria	Xanthomonadales	0.2	0.1
Proteobacteria	Deltaproteobacteria	Entotheonellales	0.1	0.3
Proteobacteria	Deltaproteobacteria	Syntrophobacterales	0.3	0.1
Proteobacteria	Deltaproteobacteria	Myxococcales	0.1	0.1
Proteobacteria	Betaproteobacteria	MND1	0.4	0.4
Proteobacteria	Betaproteobacteria	Burkholderiales	0.2	0.5
Proteobacteria	Betaproteobacteria	Burkholderiales	0.3	0.3
Proteobacteria	Alphaproteobacteria	Sphingomonadales	0.1	0.1
Proteobacteria	Alphaproteobacteria	Rhizobiales	0.2	0.4
Proteobacteria	Alphaproteobacteria	Rhodospirillales	0.7	0.1
Planctomycetes	Planctomycetia	Gemmatales	0.5	0.4
Chloroflexi	SAR202	-	0.2	0.5
Chloroflexi	-	-	0.9	0.5
Planctomycetes	Planctomycetia	Pirellulales	0.4	0.1
Nitrospirae	Nitrospira	Nitrospirales	3.5	4.7
Firmicutes	Bacilli	Bacillales	4.7	4.7
Crenarchaeota	Thaumarchaeota	Nitrososphaerales	0.7	0.6
**(B) % Taxa Differences**				
Chloroflexi	Ellin6529	-	8.7	14
Bacteroidetes	-	-	13.3	2.9
Planctomycetes	-	-	4.1	2.7
Actinobacteria	Actinobacteria	-	1.2	0.4
Actinobacteria	Acidimicrobiia	-	1.2	3.3
Proteobacteria	Gammaproteobacteria	Enterobacteriales	1.2	0
Proteobacteria	Gammaproteobacteria	Pseudomonadales	1.4	3

**Figure 5:**
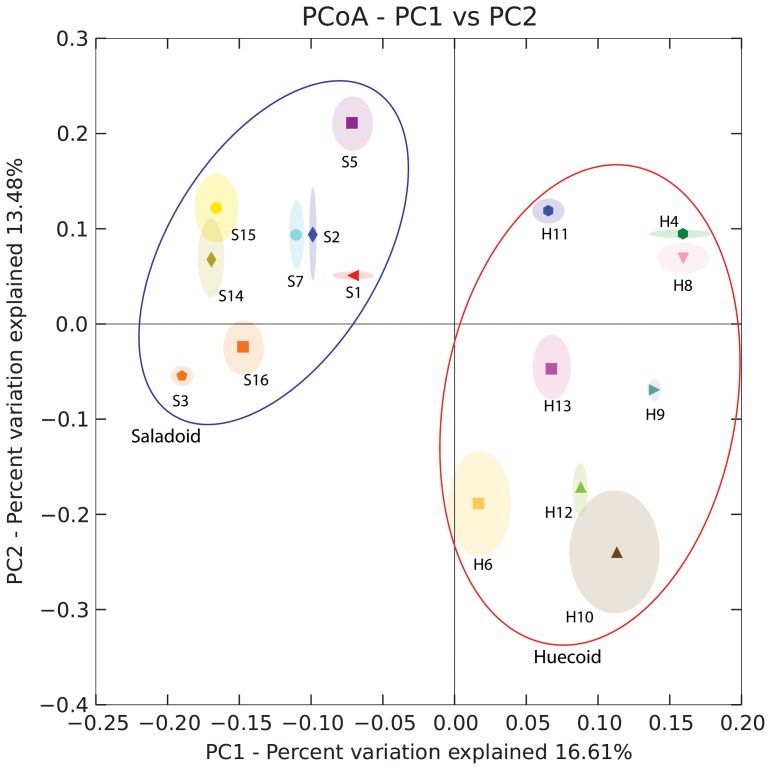
Principal Coordinate Analysis (PCoA) of the Bacterial Communities present in Huecoid and Saladoid Coprolites. Unweighted UniFrac and weighted UniFrac principal coordinates were generated and plotted using QIIME 1.8. Samples with the prefix S are from Saladoid coprolite cores and those with the prefix H were from Huecoid coprolite cores.

Figure 5 illustrates the Principal Coordinate Analysis (PCoA) of the bacterial communities found in Huecoid and Saladoid coprolite samples. PCoA of 16S rDNA sequences generated two distinct clusters, where samples originating from the same culture grouped together. Clustering of the samples may have been mainly due to the microbes described in **Table 4**.

#### 18S rDNA Analysis

**Figure 6:**
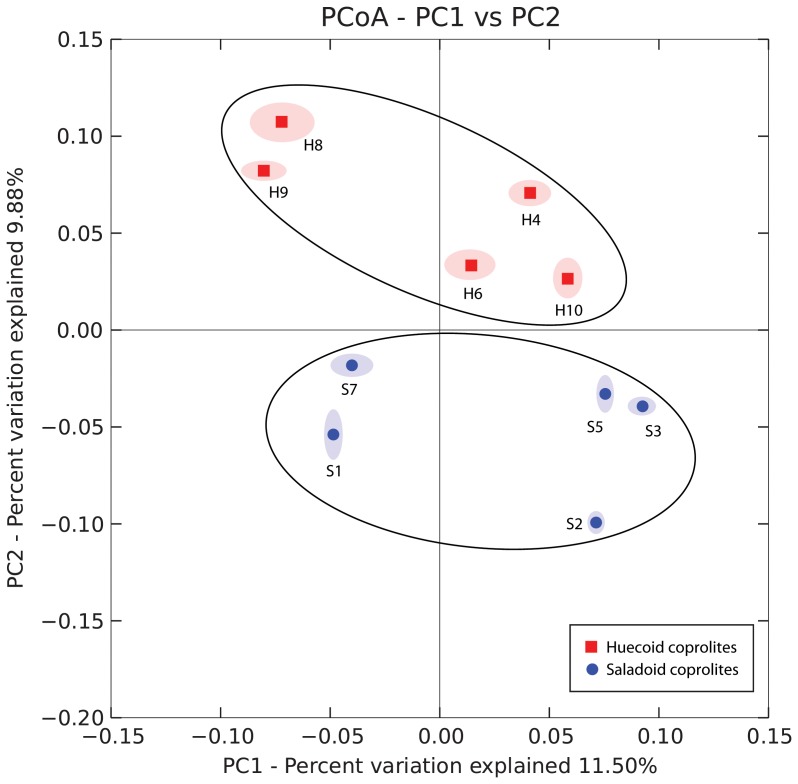
Principal Coordinate Analysis (PCoA) of the Fungal Communities present in Huecoid and Saladoid Coprolites. Unweighted UniFrac and weighted UniFrac principal coordinates were generated and plotted using QIIME 1.8. Samples with the prefix S are from Saladoid coprolite cores and those with the prefix H were from Huecoid coprolite cores.

**Figure 7:**
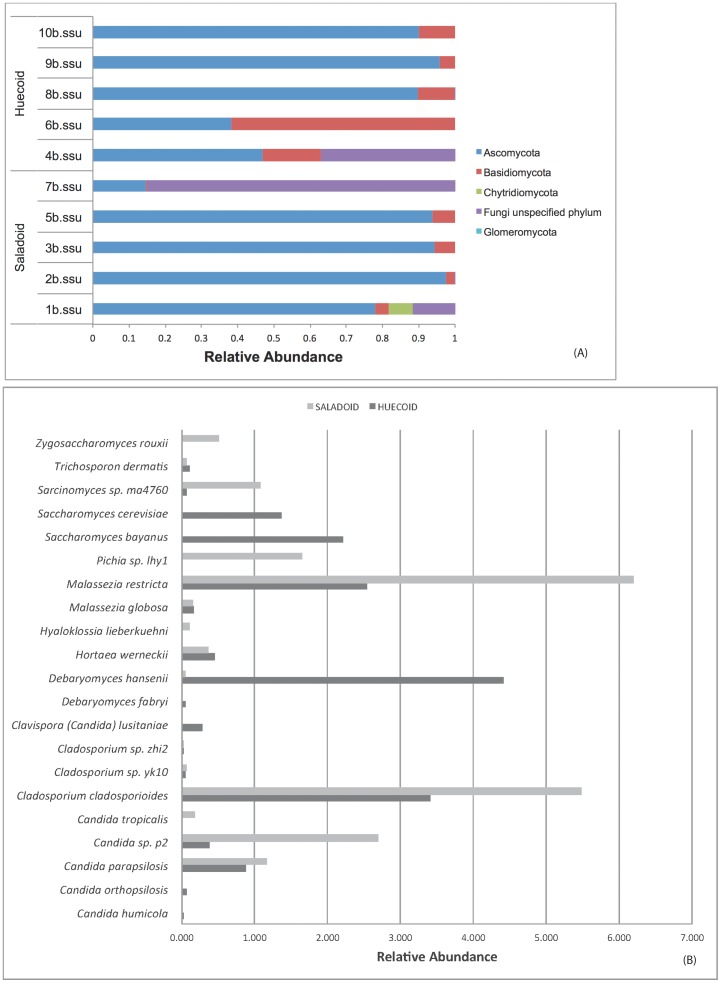
Relative abundance of fungi in coprolite samples. Panel (a) shows the proportions of fungi detected in Huecoid and Saladoid samples. Panel (b) shows the comparison of the proportions of yeasts detected in both cultures.

Figure 6 illustrates the PCoA results of the18s rDNA in Huecoid vs. Saladoid fecal microbiota. PCoA of 18S*rDNA* sequences showed two distinct clusters for the Huecoid and Saladoid samples. Figure 7 summarizes the relative abundance of fungi in coprolite samples. In general, proportions of Ascomycota were similar between both cultures. However, greater proportions of Basidiomycetes were detected in Huecoid coprolites (Figure 7a). *Saccharomyces spp.* and *Debaryomyces spp.,* were found to be more common in Huecoid feces, whereas *Candida spp*. and *Malasezia spp.* abundances were much higher in Saladoid feces (**Figure 7b**).

### Extant vs. extinct fecal microbiotas

**Figure 8:**
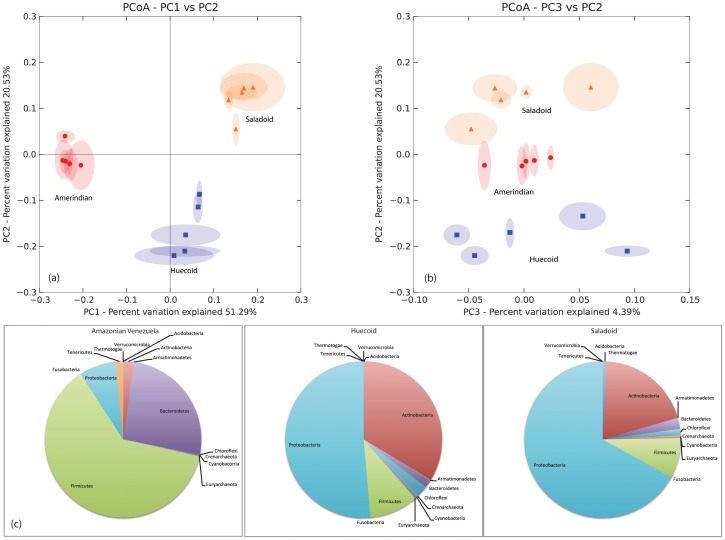
Comparison of Saladoid and Huecoid microbiota to the fecal microbiota of representative, extant indigenous cultures. Panel (a) shows the PCoA of coprolites and the fecal microbiota from extant indigenous cultures plotting PC1 vs. PC2. Panel (b) shows the PCoA of coprolites and the fecal microbiota from extant indigenous cultures plotting PC3 vs. PC2. Panel (c) shows the pie charts of taxa represented in coprolites and extant indigenous cultures.

Figure 8 compares the Saladoid and Huecoid microbiota to that of fecal microbiota from extant indigenous cultures. Panels (a) and (b) show PCoA of the three groups using PC1 with PC2 and PC3 with PC2. PC1 and PC2 (panel a) show all three groups separating on PC1, representing 51.29% of the variation. The PC3 vs. PC2 plot (panel b) illustrates the differences in microbiome composition that exist among individual samples.

Bacteroidetes were found in higher proportions in modern stool (9.03%) compared to the coprolite samples (0.49%) (**Figure 8**). Similarly, Firmicutes, Clostridiales (specifically Ruminococcaceae, Peptostreptococcaceae, Lachnospiraceae and Eubacteriaceae, among others) made up 81.00% of the modern stool microbial community, while only 18.00% of the coprolite microbiota. In contrast, Actinobacteria were more numerous (48.50% of the microbial community) in coprolites when compared to modern stool samples (2.35%). Specifically, Actinomycetales and Rubrobacterales were found to make up 28.00–33.00% and 10.00–12.00%, respectively, of the detected microbiome.

### Eukaryotic Parasites in Coprolites

Eukaryotic enteric parasites were detected in both Huecoid and Saladoid cultures (**Table 5**). There were differences, however, in parasite loads and parasite species between the two cultures. There were twice as many infected Saladoid coprolites as there were Huecoid. *Ascaris lumbricoides* and *Trichuris trichura,* were found in both cultures but with greater number among the Saladoids. *Enterobius vermicularis* and Cestodes were found also in both cultures. Hookworms were found only in Saladoid coprolites. Also, a *Paragonimus westermani* infection may have been detected in a Huecoid coprolite. The overall % positives were much higher in the Saladoid coprolites. *Dipylidium caninum* was observed in 13% of the Saladoid samples, and interestingly not in the Huecoid. Similarly, the genera *Trichostrongylus, Diphyllobotrium* as well as hookworms were found associated only with Saladoid coprolites. As part of the DNA analyses, we found fish parasites (*Goussia spp.*), which were detected exclusively in Saladoid coprolites.

**Table 5:** Enteric parasites as detected by microscopy.

Parasite Species	Huecoid (n = 8)		Saladoid (n = 7)	
	% Positive	Average Number Present	% Positive	Average Number Present
*Ascaris lumbricoides* (unfertilized)	25	56.5	43	27.0
*Ascaris lumbricoides* (fertilized)	25	151.5	43	44.7
*Trichuris trichiura*	25	57.7	57	79.3
*Enterobius vermicularis*	37.5	57.7	43	37.3
*Trichostrongylus* sp.	ND	ND	43	28.3
Hookworms	ND	ND	29	28.0
*Diphyllobothrium* sp.	ND	ND	14	26.0
*Dipylidium caninum*	ND	ND	14	30.0
Unknown Cestode	25	55.5	43	49.3
*Paragonimus westermani*	13	23.0	ND	ND
Unknown Trematode	ND	ND	14	26.0

## Discussion

Though it has been assumed (correctly in some cases), that excreta is rapidly degraded in humid tropical environments, the finding of coprolites in archaeological excavation sites located in Puerto Rico clearly contradicts this assumption. Additionally, it is very uncommon for archaeologists to focus on finding coprolites during archaeological excavations in the tropics as many are not familiar with the morphologies of the typical human or animal coprolite. If we take into consideration that feces are excreted (about 500–1,500 g/person/day), excreta should be one of the most abundant organic archeological findings at human and animal dig sites, if care is taken to search for them [17].

Microbiologically, excreta have its own biota, which, exempting periods of enteritis or other gastrointestinal diseases, and should be relatively constant in diversity and composition. Fecal microbiota are a subset of the microorganisms present in the gastrointestinal tract that are shed during defecation, and as such give much information about an individual's core gut microbiome as well as allochthonous bacteria associated with ingested food, water and very likely, air. Thus, the analysis of fossilized fecal material using NGS can be an important tool in archaeological studies to determine the prevalence of certain microorganisms, pathogenic (such as parasites, for example) and non-pathogenic alike. In terms of the overall composition of the fecal microbiome, however, differences may exist both in taxon distribution and relative abundance as a result of cultural or dietary habits. In fact, it has been clearly determined in a previous study that the fecal microbiota of these particular Antillean cultures is highly dependent on ethnicity [7].

### Analysis of the core vs. cortex of coprolites

Procrustes and Adonis analyses showed marked differences between the cores and cortices of the coprolites. Larger proportions of soil microbes (e.g. Actinobacteria) were observed in the cortices of the coprolites, which were likely due to environmental contamination, whereas smaller proportions were seen in the corresponding cores. In addition, cortices mostly shared soil microbes, possibly due to proximal burial sites, while the core microbiomes of each culture differed greatly from one another. This suggests that, although the outer parts of the coprolites were contaminated with the soil that surrounded the samples, the inner core of the coprolites remained largely intact. This is the principal reason for our downstream analyses to be conducted using only the core of the samples.

### Analysis of Huecoid and Saladoid coprolites

Both the Adonis and Permanova assays showed significant differences in the microbiome composition of coprolites from Huecoid and Saladoid sites. These analyses were performed only on samples obtained from the cores of the coprolites, thus eliminating possible differences due to environmental contamination of cortical material. These results support the hypothesis that Huecoid and Saladoid cultures, even though coexisted in the Island of Vieques, they had different cultural characteristics, most likely as a result of dietary practices.

### Key observed differences in each culture's core microbiomes: Inferences on diet

Variations observed in common gut-associated bacteria, such as Proteobacteria and Enterobacteriaceae, which were more abundant in Saladoid and Huecoid samples, respectively, suggest variations in diet or host genetics. Variations in the abundance of intestinal Proteobacteria have been associated to differences in host diet previously [20]. Differences in Bacteroides abundances were also evident between both cultures, and have been associated to a high protein diet [21]. According to the large quantities of fish bones, bivalve and crab shells found in these archaeological deposits, both cultures seemed to ingest a great amount of seafood. However, although fish bones are associated with both cultures [22], we detected freshwater fish-associated amoebic parasites (*Goussia spp*.) exclusively in Saladoid coprolites, suggesting that this particular culture may have consumed raw fish regularly. In addition, previous studies detected *Vibrio sp*. and *Debaryomyces spp* in Saladoid coprolites, further supporting this hypothesis [7]. The presence of *Paragonimus westermani* in a Huecoid coprolite implies the consumption of fresh water invertebrates, or some type of aquatic plants, the secondary hosts for this species. It is known that humans become infected by the consumption of raw food contaminated with these parasites. However, it is important to point out that this parasite is commonly confused morphologically with *Diphyllobothrium latum,* a fish-infecting parasite [23]. Interestingly, *Zea mays* (maize) was detected in coprolites from Huecoid origins, consistent with archaeological work showing its presence at the La Hueca site [24] and confirming its early presence in the Caribbean. Our results suggest that this culture may have helped introduce some of these maize strains to the Antilles during their migrations. Since they were detected in large proportions, Ascomycetes and Basidiomycetes also appear to have been important dietary elements of these cultures. However, it seems that the Huecoids had a preference for Basidiomycota fungi. Although these conclusions are highly hypothetical and perhaps speculative, we believe this is a good starting point if we are to compare future studies such as the one carried out here.

### Enteric Parasite infections

The greater parasite load observed in Saladoid coprolites suggests a difference in living arrangements, whereby the population was more likely to be exposed to fecal material and thus parasite transmission. Parasite analyses were done microscopically in a separate laboratory, however the results also show major differences between the taxonomic compositions present in both cultures. A wider variety of parasitic species was detected in the Saladoids, which could also be associated to the way this culture handled their food (as previously mentioned). It is also known that both cultures had dogs as pets, particularly the Saladoids. We detected the canine parasite *Dipylidium caninum* in Saladoid samples, also suggesting pet ownership and perhaps very close contact. However, little if anything is known about the interactions of the Huecoids with dogs. Dogs have a tendency to eat human feces, and this may be one manner in which the parasites are passed from person to person, with the dogs being the vectors. The presence of *Dipylidium caninum* supports this hypothesis, as does the high prevalence of most intestinal parasites detected in the Saladoid coprolites. In addition, present-day ethnic groups in the Amazon basin maintain close relations with dogs and even share their living space with these animals, and although highly speculative, the presence of zoonotic parasites in the Saladoid coprolites is intriguing, and requires further analysis. It is also intriguing that well-formed coprolites had such a high prevalence of enteric parasites, since many of these pathogens result in enteritis in present-day populations, which would not be amenable to the formation of coprolites. It is thus possible that multiple infections with parasites were common, and yet these infections failed to cause enteritis.

The observed differences, both in the core fecal microbiota and the detected remnant food, suggest major variations in the diets of these extinct cultures. This is further supported by the diversity and relative abundances of the parasites detected in each culture. The Saladoid and Huecoid deposits in Vieques are separated by a distance of 15–150 m [7], which suggests that the observed variations in fecal microbiota are not of geographical origins but more likely due to cultural and dietary differences.

### Modern Amazonian stool vs. ancient Antillean stool: Considering the effects of taphonomic conditions

The microbiota detected in the coprolites was associated with those found in modern stool, but mainly differed in the proportions observed for each phylum. We observed two main phyla of Gram-positive bacteria in our samples (Actinobacteria and Firmicutes) and two main phyla of Gram-negative bacteria (Bacteroides and Proteobacteria).

As suggested by other studies, Gram-positive bacteria tend to have a high resistance towards dry conditions, so the presence of such a diverse array of these microorganisms preserved in these coprolites was expected [25], [26]. Actinobacteria are notorious for their resistance to arid conditions; this resilience towards taphonomic conditions makes them more likely to be detected in high abundances in archaeological samples [27]. In terms of what can or cannot be detected, we can only assume that over centuries or millennia, most, if not all of the microbiota in the coprolites has been inactivated and, unless there is rapid dehydration, the cells will be lysed and the free DNA will be rapidly degraded. It has been observed that naked DNA will remain relatively undegraded for very short periods of time in aquatic environments [18], [19], however, DNA conserved intracellularly in (for example), dormant microorganisms may most likely be better preserved against taphonomic conditions than naked DNA. Again, the mere presence of coprolites indicate that there was rapid dehydration, and thus protection of the nucleic acid material from any extracellular nucleases.

Compared to modern stools, the percentage of Firmicutes detected in the coprolites was much lower. Firmicutes are known to have a low G/C content in their genomes, possibly allowing for faster DNA degradation throughout the fossilization process when compared to bacteria with high [G/C], as in the case of the Actinobacteria, (Taxonomy Browser, NCBI). Interestingly, in spite of *Clostridium* being spore-formers, the proportion of Firmicutes in coprolites was much lower than detected in the modern stool. However, this may be linked to previous observations suggesting a higher presence of vegetative cells when *Clostridium* is located in the human gut as compared to soil environments, where the conditions are stressful [28]. In addition, these cells are highly sensitive to oxygen and rapidly die when exposed to oxic conditions. Also, some bacterial spores tend to germinate when they come in contact with the gastrointestinal environment [29] thus leading to more vegetative cells rather than spores.

Low levels of *Bacteroides* were detected in coprolites compared to modern stool, however, this could be due to the strict anaerobes high sensitivity to oxygen. Interestingly, when compared to modern feces the coprolites showed a higher proportion of Proteobacteria in the microbial community. Though initially counter-intuitive, it is now known that dormancy is a common strategy for long term survival when Gram-negative microorganisms are faced with nutrient and water starvation, exposure to radiation and drastic changes in temperature. Nutritional stress, for example, has been shown to induce the transcription of stress proteins, which ultimately convey the cell a higher resistance towards variations in these abiotic factors [28], [30]. This starvation-induced multi-stress resistance, and other bacterial dormancy mechanisms have been characterized, including a spontaneously initiated dormancy as well [31]. This dormancy state appears to be reversible in some cases [32], [33], [34] (though the mechanisms for resuscitation remain largely unknown), and could have played a factor in the recent isolation of viable microorganisms from coprolite samples [35].

Although these latent bacteria could account for the diversity of Gram-negative bacteria observed in the coprolites, another possibility could be that the relative half-life of these microorganisms is much higher than that of those no longer detectable in the sample. Depending on their half-life, low abundance taxa (or rare species) could have been eliminated from the sample whilst taxa initially present in higher concentrations could still be detectable after preservation for a thousand years.

### Differences in coprolite microbiota and the ‘Huecoid problem’

The discovery of the Huecoid culture in the 1970s by Chanlatte and Narganes led to formulation of the ‘Huecoid problem’, the question whether the Huecoids were ethnically distinct from the Saladoids or were simply a Saladoid subgroup. Classical archeology has provided much evidence for cultural distinctness between the two groups, but has not led to a resolution of the problem. There are many material ways in which the Huecoid and Saladoid cultures may be separated [36]. Technological differences include marked differences in pottery and lapidary carvings and more subtle differences in stone tools such as Adzes and Celts. The Saladoids apparently preferred to make their adornments in mollusk conch, in contrast the Huecoids preferred stone. Evidence of dietary differences includes the absence of turtle remains in Huecoid settlements implying they were taboo [22]. Evidence of religious differences are abundant. Firstly, burial practices are distinct, with Saladoid interments occurring within their settlements, while Huecoid burials have yet to be encountered. Iconography is distinct, unique Huecoid symbolism is seen with birds interpreted as condors, carrying human heads, not represented in Saladoid lapidary or ceramics [24]. The range of materials from which Huecoid lapidary was fashioned is much more diverse than Saladoid as are the decorative themes. Huecoid settlements have been associated with increased ritual activity, indicated by an increased incidence of implements associated with what appears to be an early version of the cohoba ritual [37], [38]. Thus, the Huecoids have been described as ‘religious specialists’, with a spiritual role servicing the majority Saladoid community [24] (see Pagan-Jimenez 2007 for a summary). However, while physical evidence of cultural distinctness is abundant, the true role of the Huecoids remains elusive. Ancient DNA studies might hold the key to answering the ‘Huecoid problem’. In our study, we have addressed the question from a unique angle, that is, of using paleomicrobiomics. Our results clearly show that the gut microbiota, prokaryotic and fungal, were distinct between the two cultures. While there exists a possibility that these differences might reflect differences in mammalian host genetics [39], [40], which would indicate ethnic uniqueness, we interpret the differences to be mainly due to diet, and, perhaps to interaction with pets, as in the case of enteric parasites.

Given the apparent difference in diet indicated by differences in the intestinal microbiota, this allows us traction into the Huecoid problem. There are very few examples of groups that are differentiated by diet and live in conjoined settlements, but are still part of the same ethnic identity. Thus, it is hard to find a modern parallel of the scenario proposed by Rouse that the Huecoids were a Saladoid subgroup [41]. This does not rule out some unique arrangement within Saladoid society, and so while we would propose that both archaeological and paleomicrobiomic data strongly suggest a distinct ethnic identity to the Huecoids, this awaits confirmation.

## Conclusions

We successfully extracted and sequenced DNA from archaeological fecal samples in order to assess possible differences in the fecal communities of individuals from Saladoid and Huecoid indigenous cultures. Our data show that, contrary to common belief, the formation and preservation of coprolites and DNA contained in these coprolites under humid, tropical environments for thousands of years is possible. Not only is the DNA still present, it was also detected by PCR amplification and sequenced successfully. We also demonstrate a clear difference between the fecal microbiota of these two cultures, and therefore variations in terms of their diet and/or genetic heritage. Similar to previous results, our data also supports the hypothesis stating that the Huecoids and Saladoids originated and migrated independently from their respective origins, as opposed to having a common ancestry.

This study is one of the first in its kind and we hope will point to the importance of coprolites as important cultural markers and thus any archaeological dig should include the search and preservation of any coprolites found at the sites. This study underlines the importance of such samples for future paleomicrobiological studies. The results have several implications. First, it confirms that coprolites are not completely degraded in humid, tropical environments and thus can be formed under suitable taphonomic conditions. Second, it implies that dietary and/or host genetic differences in ancient populations may be reflected in differences in gut microbiome composition and it confirms that the two indigenous cultures were indeed distinct. Third, it demonstrates that paleomicrobiomics could be a powerful tool to assess dietary, health, genetic and cultural differences between ancient populations. Finally, it implies that these two cultures retained distinct technological/cultural differences during a period of close proximity and peaceful co-existence and suggests that the two populations, at least at this location, may have contributed to form the latter day Taínos, the Amerindians present at the point of Columbian contact.
